# First vacuum-assisted excision of a breast intraductal papilloma in the pediatric age group: Case report

**DOI:** 10.1016/j.radcr.2024.08.100

**Published:** 2024-09-17

**Authors:** Reem Ahmed Al Mazrouai, Umaima al Wahaibi, Fathiya al Rahbi, Badriya al Qassabi, Suaad alaghbari

**Affiliations:** a Sultan Qaboos Comprehensive Cancer Care and Research Centre (SQCCCRC), Muscat, Oman; b Oman Medical Specialty Board (OMSB), Muscat, Oman; c Sultan Qaboos University Hospital, Muscat, Oman

**Keywords:** Intraductal papilloma, Breast tumors in pediatric group, Vacuum-assisted excision, Surgical excision

## Abstract

Intraductal papillomas (IP) are benign breast tumors that can occur in adolescents and young women, but they are extremely rare in pediatric age group and their occurrence in pediatric patients is not well documented in the medical literature [1,2].

The standard approach for IPs in teenagers involves conservative management with careful monitoring and follow-up imaging. However, in select cases, surgical intervention may be warranted to confirm the diagnosis and prevent complications such as bleeding or infection [3,4].

A novel, less invasive alternative to surgical excision is ultrasound-guided vacuum-assisted excision (VAE), which uses ultrasound to accurately target and extract the lesion using a vacuum-assisted device [4,5]. Compared to surgical excision, VAE offers the advantage of being a less invasive procedure, which leads to a decrease in the number of complications. Over the past 10 years, this method has become increasingly popular due to its ability to specifically and efficiently remove intraductal papilloma while minimizing risks and preserving the structure of the breast [5,6].

To our knowledge, this is the first documented use of VAE in a pediatric patient, as demonstrated in our case of a 9-year-old with nipple discharge successfully managed with VAE, highlighting its potential as a viable treatment option for pediatric breast lesions. This case highlights the potential use and success of VAE as a management option for breast lesions in pediatric patients. Further research and additional case reports are needed to further establish the efficacy and safety of this technique in this specific age group.

## Case report

A 9-year-old girl presented to surgical outpatient clinic in June 21, 2021, accompanied by her parents. She complained of spontaneous nipple discharge on the right side for a month. The discharge was brownish to greenish in color and has significant impact on the child's daily life, including her school activities, where her clothes became soaked. This was the first time she had experienced such discharge, and she had no history of breast disease or any previous breast trauma. There was also no family history of breast or ovarian cancer.

During the physical examination of the right breast, there was an elongated firm lump measuring 2.5 × 1 cm at 9 o'clock. The lump was mobile and not tender, and no nipple discharge was observed. There were no palpable swollen lymph nodes in the armpit area.

Laboratory investigations were conducted, including a complete blood count (CBC), coagulation profile, and hormonal assessments. All results were within normal ranges, encompassing estrogen, progesterone, prolactin, luteinizing hormone (LH), and follicle-stimulating hormone (FSH) levels.

**Fig. 1:**
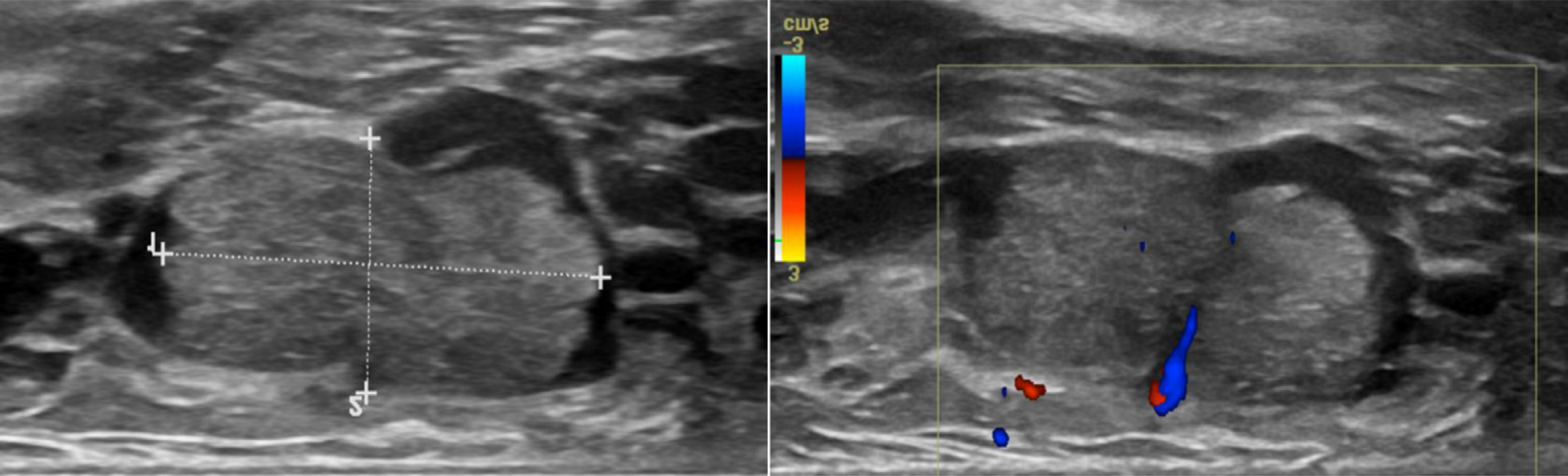
Greyscale ultrasound examination shows intraductal homogeneous solid lesion at 9:00 peri areolar region measuring 2.3 × 1.7 × 1.2 cm. Note the dilated anechoic duct containing the lesion. Color Doppler shows internal vascularity within the lesion.

Imaging investigations including a breast ultrasound examination was performed. The ultrasound of the right breast showed notable duct ectasia (an expansion of the milk ducts) with a large intraductal lesion at 9 o'clock measuring 2.3 × 1.7 × 1.2 cm, which was likely a papilloma (see Fig. 1).

**Fig. 2:**
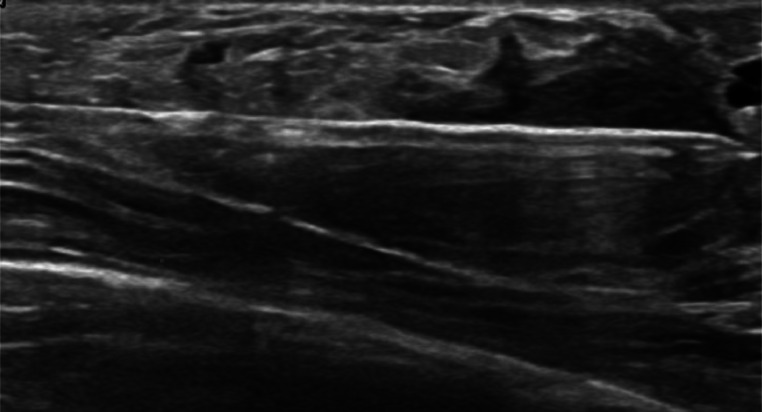
The image demonstrates the utilization of ultrasound guidance and a 14-gauge core biopsy needle to accurately target the solid part of the lesion during the biopsy procedure.

Based on the ultrasound findings, the mass classified as BI-RADS category 4a, which indicates a low level of suspicion per the Breast Imaging Reporting and Data System (BI-RADS). Consequently, a biopsy of the suspected intraductal papilloma was performed under ultrasound guidance using local anesthesia. A 14-gauge core biopsy needle was used to obtain 4 complete core samples (see Fig. 2). The biopsy aimed to confirm the diagnosis and check for any complex features or atypical characteristics.

Histopathological examination showed enlarged ducts, some of which had surrounding swelling and mild inflammation. A solitary papillary-like projection without a fibrovascular core was noted in a few of these ducts. There were no signs of a true papillary lesion, atypia, in situ abnormalities, or invasive malignancy.

Following discussions with the surgeon, pathologists, radiologist, and the patient's family, it was decided that the patient would have follow-up ultrasounds every 6 months. In these follow-up scans, The lesion showed interval growth in size. The lesion exhibited internal blood flow, but no new lesions were detected. Given these developments and the ongoing impact of the discharge on the patient's daily life and school activities, the decision was made to proceed with an ultrasound-guided vacuum excision. This approach was chosen to ensure precise diagnosis and effective management of the condition.

The parents were advised that this procedure is considered a daycare operation, typically lasting between 40 to 60 minutes, and serves as a less invasive alternative to surgical excision, which poses risks of disrupting breast anatomy, particularly in pediatric patients.

**Fig. 3:**
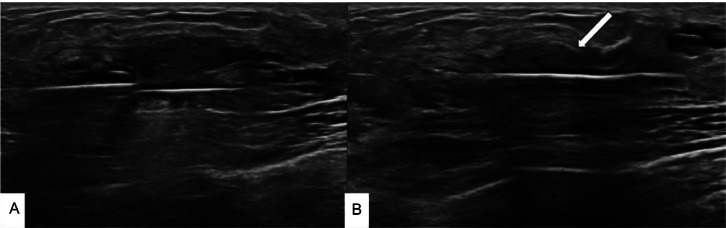
The provided images depict the utilization of a 7-gauge core biopsy needle for vacuum excision. In the image labeled (A) you can observe the needle positioned at the base of the lesion. In the image labeled (B) you can notice the gradual flattening of the lesion until it is completely excised (white arrow).

Once consensus was reached, the procedure was conducted in the radiology intervention suite under general anesthesia, chosen to prevent potential difficulties with the child's cooperation. A 7-gauge core biopsy needle was utilized for the vacuum excision, and the intraductal papilloma was entirely removed in fewer than 10 samples (Fig. 3).

During the procedure, a complication arose in the form of a ductal skin fistula, which led to fluid leakage. This issue was promptly managed by applying compression for 30 minutes, effectively controlling the leakage. The patient recovered well from the anesthesia postprocedure, with the breast appearing normal and no evidence of hematoma or ongoing leakage. The dressing remained dry when checked 30 minutes later.

At a subsequent follow-up visit at the outpatient clinic, both the patient and her parents expressed satisfaction with the outcomes of the procedure. Additionally, the initial symptom of nipple discharge that led to the intervention had ceased, marking a successful resolution of the issue.

**Fig. 4:**
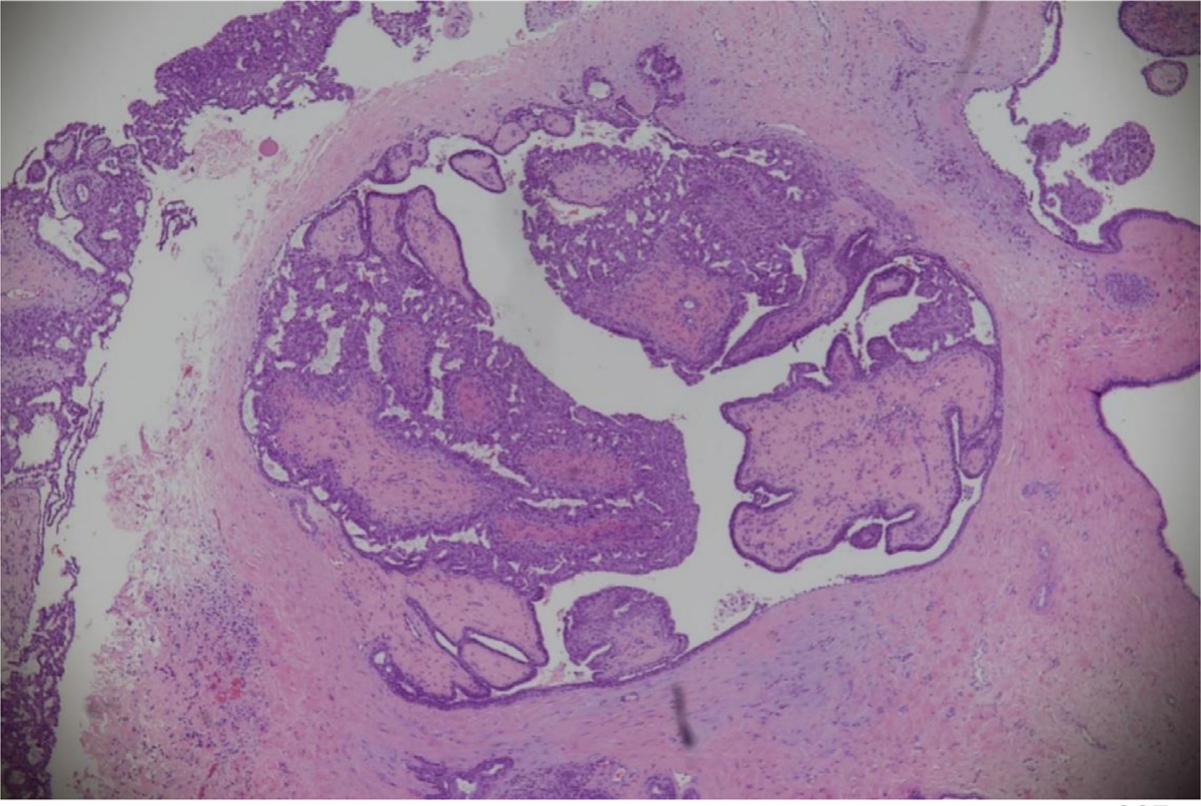
(hematoxylin and eosin stain, ×200): Dilated duct containing arborizing fronds of tissue with well‐developed central fibrovascular cores covered by epithelial cells and overlying myoepithelial cells. Foci of epithelial hyperplasia is seen.

**Fig. 5:**
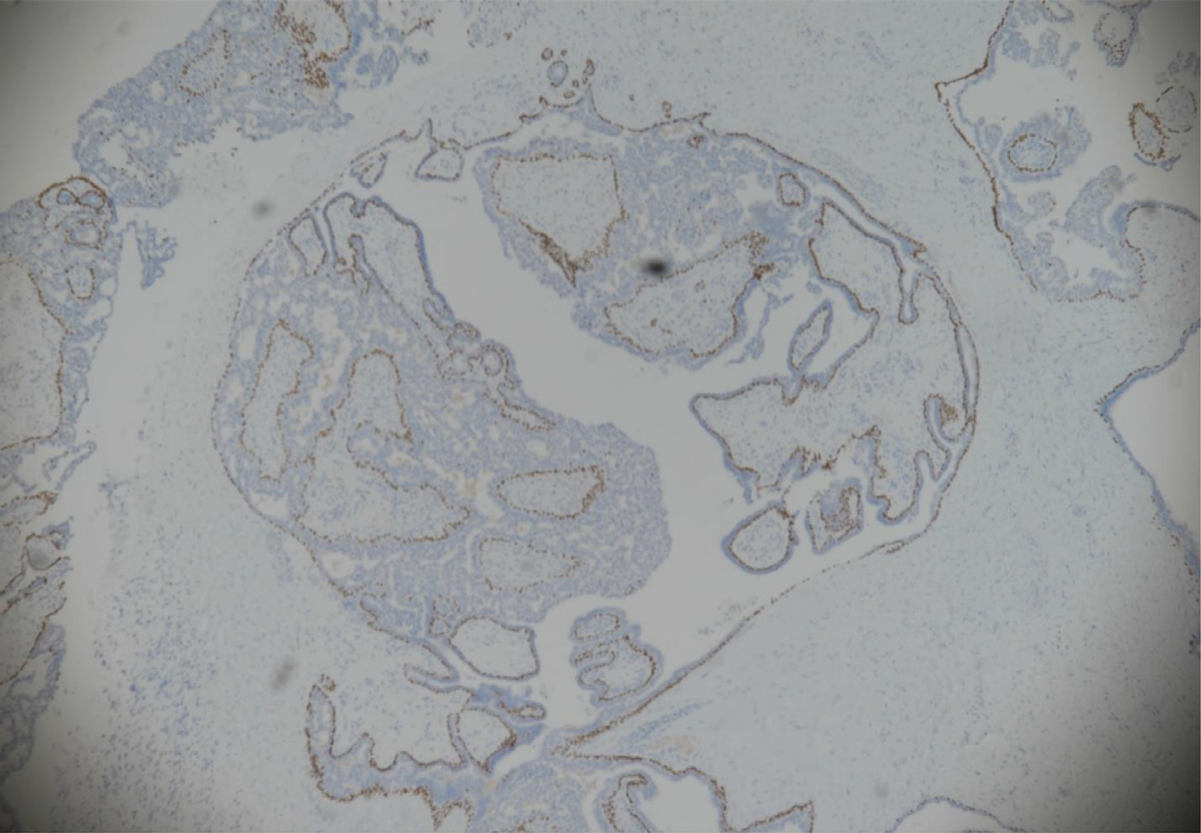
(P63 immunohistochemistry stain): P63 immunostain highlighted the myoepithelial layer in the dilated ducat and fibrovascular cores.

**Fig. 6:**
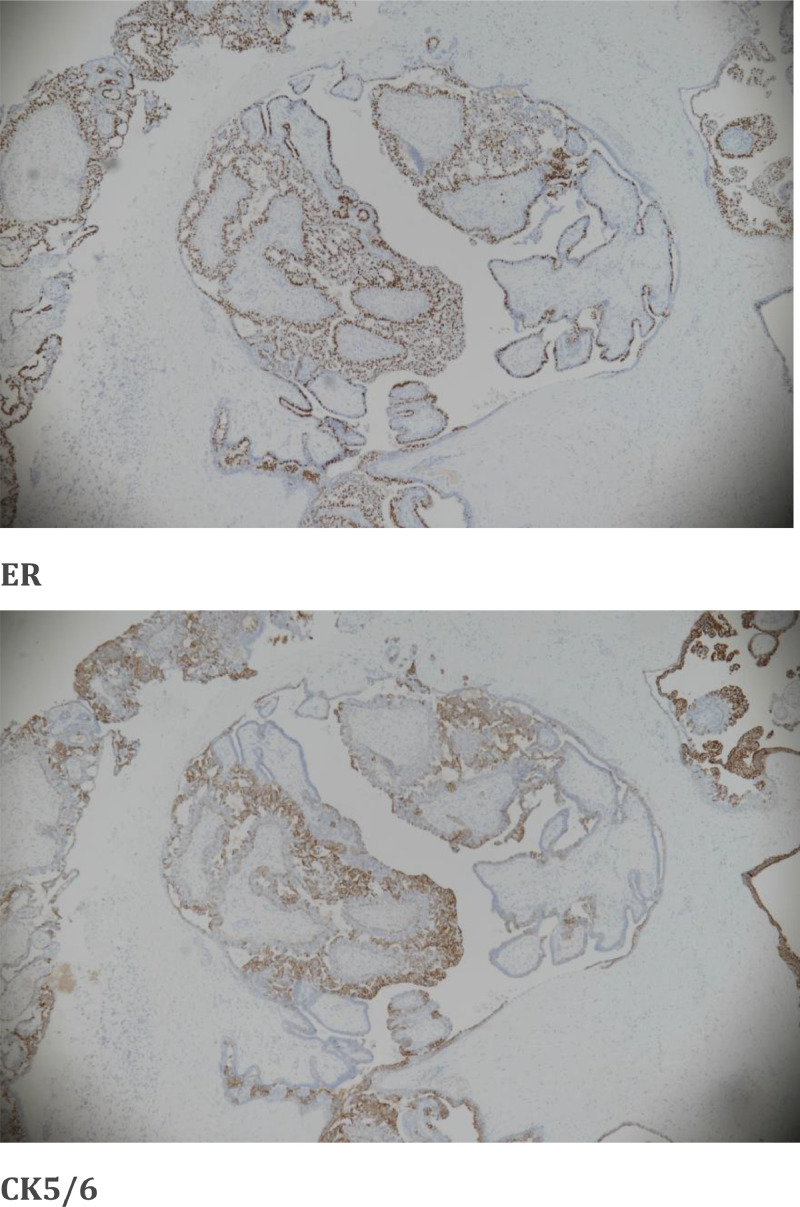
(A and B) (ER &CK5/6 immunohistochemistry stain):). ER showed heterogeneous nuclear stain and CK5/6 showed patchy mosaic pattern, supporting the diagnosis of usual ductal hyperplasia.

The results of the histopathology examination showed dilated cystic spaces containing fibrovascular network in the center covered by outer epithelial and myoepithelial cells (Fig. 4) with frond formation in the cystic space. The epithelia demonstrated usual hyperplasia, which connected with each other to form complex tubular and cribriform structures. P63 immunostain highlighted the myoepithelial layer in the dilated ducat and fibrovascular cores (Fig. 5). ER showed heterogeneous nuclear stain and CK5/6 showed patchy mosaic pattern, supporting the diagnosis of usual ductal hyperplasia (Figs. 6A and B). No DCIS or malignancy detected in the submitted sample.

Furthermore, a subsequent follow-up ultrasound examination confirmed the resolution of the previously observed intraductal papilloma. The imaging showed no signs or evidence of the papilloma that had been previously detected.

Based on these positive outcomes and the absence of any further concerns, the patient was discharged from the clinic without the need for additional follow-up appointments.

Oral and written consent were taken from the patient for publication purposes.

## Discussion

Intraductal papillomas are benign tumors that originate from the milk ducts in the breast. While they are rare in children and teenagers, the exact incidence of this condition in this age group is not well-established. These tumors are more commonly seen in women over the age of 40, although they can occur in adolescents and young women as well [1,2,7].

In children, IP are usually associated with hormonal imbalances or genetic disorders that affect breast development. Occasionally, they may also be associated with other benign breast conditions like fibroadenomas or cysts [8].

The management of IP in children follows similar principles to that in adults and may involve the excision of the lesion to confirm the diagnosis and prevent complications such as bleeding or infection. However, due to the rarity of these tumors in pediatric patients, there is limited information available regarding their management and long-term outcomes in this population [[7], [8], [9]].

However, it is important to note that surgical interventions in the breast for pediatric patients are generally not recommended unless medically necessary, as they can disrupt normal breast development. Breast surgery in this age group may lead to long-term cosmetic or functional issues, and the risks of infection, bleeding, and scarring are also associated with these procedures [8,9].

Vacuum-assisted excision (VAE), a less invasive alternative, facilitates the removal of IPs while conserving the breast's ductal structure. This method is advantageous because it is minimally invasive, permits outpatient procedures, minimizes scarring, and shortens recovery times compared to traditional surgery [4,5].

The VAE procedure involves the insertion of a specialized instrument through a minimal incision, using vacuum technology to excise the lesion, thereby preserving ductal integrity and minimizing risks like bleeding and infection [5,6].

There is limited documentation in the literature concerning biopsy-confirmed intraductal papilloma specifically in the pediatric and teenage populations. Among the reported cases, surgical excision was consistently utilized as the preferred approach for management.

One case involved a 12-year-old girl who presented with bloody nipple discharge that was managed by excisional biopsy and histological examination confirmed a diagnosis of intraductal papilloma [10].

Another case involved an adolescent girl presented with nipple discharge and was found to have an intraductal lesion in ultrasound. biopsy showed partially sclerosed intraductal papillomas without atypia or malignancy. The lesion was also treated by surgical excision [11,12].

In summary, the management of IPs in children and adolescents should be tailored to individual cases, considering factors such as the size and location of the lesion, the patient's age and overall health, and the presence of high-risk features. VAE is a potential treatment option in this population, but each case should be carefully evaluated to minimize risks and ensure the best possible outcomes.

## Conclusion

Our case report presents the first documented case of vacuum-assisted excision being performed on a patient as young as 9 years old with an intraductal papilloma. This is a significant finding as there is limited information available on the use of this technique at such young age groups. The successful management of the intraductal papilloma using vacuum-assisted excision highlights the potential benefits of this minimally invasive approach in pediatric patients. Further research and additional case studies are needed to better understand the efficacy and long-term outcomes of vacuum-assisted excision in this specific age group.

## Patient consent

Informed consent for the ultrasound-guided vacuum-assisted excision (VAE) procedure was obtained from the patient's guardians. Given the patient's age, consent was secured from her parents after providing them with detailed information about the procedure, its risks, and benefits. The guardians provided written consent prior to the initiation of the procedure, acknowledging their understanding and agreement to proceed with the minimally invasive treatment.
